# Supplementary material for: Simple Application and Adherence to Gout Guidelines Enables Disease Control: An Observational Study in French Referral Centres
Source: J Clin Med. 2022 Sep 28;11(19):5742. doi: 10.3390/jcm11195742 (PMC9570816; doi:10.3390/jcm11195742)
Supplement: Supplementary file 1 [file jcm-11-05742-s001.zip › jcm-1914813-supplementary.pdf]

Table S1: Patient baseline characteristics, per centre.

**Baseline characteristics**	**Bichat**	[a]	**Lariboisière**	[a]	**Saint Philibert**	[a]
Demographics						
Male	71 (71%)	0	89 (89%)	0	83 (83%)	0
Age at inclusion (years)	65.4 ± 13.1	0	57.1 ± 16.5	0	65 ± 14.9	0
Body mass index (kg/m^2)	28.3 ± 5.6	17	27.3 ± 4.3	25	28 ± 4.7	11
Ethnicity		0		9		0
Caucasian	38 (38%)		43 (47.3%)		82 (82%)	
North African	20 (20%)		19 (20.9%)		10 (10%)	
Sub Saharan African	29 (29%)		15 (16.5%)		4 (4%)	
Southeast Asian	7 (7%)		5 (5.5%)		3 (3%)	
Middle East	0 (0%)		2 (2.2%)		0 (0%)	
Others	6 (6%)		7 (7.7%)		1 (1%)	
Socio-economic status		3		23		12
Manager	0 (0%)		7 (9.1%)		7 (8%)	
Academic profession	8 (8.2%)		18 (23.4%)		13 (14.8%)	
Worker, farmer	14 (14.4%)		9 (11.7%)		12 (13.6%)	
Unemployed	8 (8.2%)		4 (5.2%)		1 (1.1%)	
Home keeper, retired, disabled	67 (69.1%)		39 (50.6%)		55 (62.5%)	
Beverage intake		0		1		0
No beverage consumption	75 (75%)		61 (61.6%)		58 (58%)	
Current alcoholic intoxication[b]	15 (15%)		15 (15.2%)		25 (25%)	
Former alcoholic intoxication	8 (8%)		7 (7.1%)		11 (11%)	
Current excessive sweetened beverage intake[c]	1 (1%)		3 (3%)		3 (3%)	
Former excessive sweetened beverage intake	0 (0%)		3 (3%)		0 (0%)	
Current alcoholic intoxication and excessive sweetened beverage intake	1 (1%)		9 (9.1%)		3 (3%)	
Former alcoholic intoxication and excessive sweetened beverage intake	0 (0%)		1 (1%)		0 (0%)	
Smoking status		0		1		0
No	59 (59%)		71 (71.7%)		60 (60%)	
Current smoker	16 (16%)		13 (13.1%)		12 (12%)	
Former smoker	25 (25%)		15 (15.2%)		28 (28%)	
Diet high in purine and/or fructose	43 (57.3%)	25	36 (43.9%)	18	37 (41.1%)	10
Practice of regular physical activity	8 (10%)	20	17 (20.5%)	17	6 (7.1%)	16
Rheumatologic and non-rheumatologic comorbidities						
Osteoarthritis	23 (23%)	0	17 (17%)	0	32 (32%)	0
Diabetes mellitus	32 (32%)	0	25 (25.3%)	1	27 (27%)	0
High blood pressure	71 (71%)	0	46 (46.5%)	1	64 (64%)	0
History of major cardiovascular event (stroke, myocardial infarction, lower limb arteriopathy)	21 (21%)	0	17 (17.1%)	1	28 (28%)	0
Congestive heart failure	14 (14%)	0	8 (8.1%)	1	16 (16%)	0

Dyslipidaemia	30 (30%)	0	21 (21.2%)	1	49 (49%)	0
Liver disease	2 (2%)	0	5 (5.1%)	1	49 (49%)	0
Obesity (body mass index > 30 kg/m^2)	31 (37.3%)	17	18 (24%)	25	25 (28.1%)	11
Family history of[d]		0		0		0
Gout (first or second degree)	5 (5%)		26 (26%)		24 (24%)	
Renal colic (first or second degree)	0 (0%)		4 (4%)		1 (1%)	
Hyperuricaemia (first or second degree)	0 (0%)		2 (2%)		0 (0%)	
Background treatments for comorbidities						
≥2 hyper-uricaemic treatments[e]	56 (56%)	0	34 (34.3%)	1	36 (36%)	0
≥2 hypo-uricaemic treatments[f]	36 (36%)	0	21 (21.2%)	1	21 (21%)	0
End-stage kidney failure treatment (dialysis, kidney transplant)	2 (2%)	0	2 (2%)	1	0 (0%)	0
Gout characteristics						
Gout duration (years)	3.1 ± 5.7	9	5.9 ± 8.8	2	8.3 ± 9.7	3
Number of flares in the 6 months before baseline	1 [1 ; 2]	5	1 [1 ; 2]	5	1 [1 ; 3]	8
At least one gout complication at baseline	84 (84%)	0	68 (68%)	0	77 (77%)	0
If yes, type[d]		0		0		0
Ultrasound or subcutaneous tophi	45 (53.6%)		36 (52.9%)		63 (81.8%)	
Renal lithiasis on imaging and/or renal colic	2 (2.4%)		11 (16.2%)		22 (28.6%)	
Chronic kidney disease (CKD 3 and above)	60 (71.4%)		37 (54.4%)		31 (40.3%)	
Gouty arthropathy	15 (17.9%)		21 (30.9%)		22 (28.6%)	
On-going urate lowering therapy (ULT)	17 (17%)	0	31 (31%)		33 (33%)	0
If yes, ULT drug		0		0		0
Allopurinol	8 (47.1%)		16 (51.6%)		21 (63.3%)	
Febuxostat	9 (52.9%)		14 (45.2%)		11 (33.3%)	
Benzobromarone, Probenecid	0 (0%)		1 (3.2%)		1 (3%)	
Lesinurad	0 (0%)		0 (0%)		0 (0%)	
Rasburicase, Pegloticase	0 (0%)		0 (0%)		0 (0%)	
Bitherapy	0 (0%)		0 (0%)		0 (0%)	
Serum urate level (mg/dL)	84.5 ± 23.5	5	80.6 ± 24.2	8	80.3 ± 23.4	4
First visit context:		0		0		0
Out-patient referral	32 (32%)		92 (92%)		66 (66%)	
In-hospital care	68 (68%)		8 (8%)		34 (34%)	
Patient specifically referred to expert centre	79 (79%)	0	39 (39%)	0	60 (60%)	0
If yes, reason		0		0		0
From primary care for treatment initiation	4 (5.1%)		1 (2.6%)		4 (6.7%)	
From another hospital department for gout management	68 (86.1%)		30 (76.9%)		42 (70%)	
Non-control at a submaximal dose of ULT in primary care	0 (0%)		3 (7.7%)		5 (8.3%)	
Non-control at a maximal dose of ULT in primary care	1 (1.3%)		2 (5.1%)		2 (2.3%)	
Non-control with non-referred management	0 (0%)		0 (0%)		1 (1.7%)	
For initial hospital prescription	0 (0%)		0 (0%)		1 (1.7%)	
Already follow in the expert centre for another rheumatological pathology	5 (6.3%)		1 (2.6%)		3 (5%)	
From primary care, for diagnosis re-evaluation	1 (1.3%)		0 (0%)		1 (1.7%)	

From primary care, for re-evaluation because of tolerance difficulties to standard ULT	0 (0%)	0 (0%)	1 (1.7%)
For personal convenience	0 (0%)	2 (5.1%)	0 (0%)

Mean ± SD, median [Q1;Q3], n (%).

[a]Missing values (n)

[b]More than 21 units of alcohol per week in men, and more than 14 per week in women (OMS recommendations)

[c]At least one serving per day [17]

[d]Multiple choice

[e]Beta-blocker, diuretics, aspirin

[f]Losartan, calcium channel blocker, atorvastatin, fenofibrate, ezetimibe

Table S2: Lost to follow-up patients characteristics.

Baseline characterics of patients lost to follow-up just after baseline (M0)	
	Effective (n, %) or mean ± SD
Baseline meeting context (%)	
Consultation	32 (48.5%)
Hospitalization	34 (51.5%)
Age at inclusion (years)	63.6 ± 13.4
Age at gout diagnosis (years)	59.7 ± 15.5
Duration of disease before inclusion (years)	4.2 ± 6.6
Gout flares over the last 6 months before inclusion	1.9 ± 2.4
M0 uricemia (mg/L)	8.09 ± 2.54
Gout complications already present before M0	1 ± 0.8
Number of extra-rheumatologic comorbidities	1.8 ± 1.5
ULT prescribed at the end of the M0 consultation	57 (86.4%)
Allopurinol	24 (42.9%)
Febuxostat	32 (57.1%)

Reason for not attending a visit in the follow-up			
		Effective (n, %)	
	M6	*M12*	*M24*
Visit status			
- honored	205 (68.3%)	161 (53.7%)	122 (40.7%)
- non-honoured	95 (31.7%)	139 (46.3%)	178 (59.3%)
CH Saint Philibert	14 (14.7%)	37 (26.6%)	52 (29.2%)
CHU Bichat	47 (49.5%)	60 (43.1%)	78 (43.8%)
CHU Lariboisière	34 (35.8%)	42 (30.2%)	48 (27.0%)
If non-honoured, reason:			
- unknown	62 (65.3%)	79 (56.8%)	114 (64%)
- patient’s decision	10 (10.5%)	10 (7.2%)	12 (6.7%)
- voluntary non-recall from the rheumatologist	11 (11.6%)	27 (19.4%)	25 (14%)
- intercurrent health problem	12 (12.6%)	23 (16.5%)	24 (13.5%)
- death	0 (0%)	0 (0%)	3 (1.7%)

M0: baseline; ULT: urate lowering therapy.

Table S3: Subgroup analysis to compare the "lost to follow-up just after baseline" to the "lost after at least two consultations" patients profile (n = 300).

	Missing data (n)	***"lost to follow-up" after M0 (n = 66)***	***M0 and at least one another consultation (n = 234)***	***p-value***
ULT prescribed at the end of the M0 consultation (n, %)	0	57 (86.4%)	197 (84.2%)	0.81
Allopurinol		24 (42.9%)	102 (53.1%)	
Febuxostat		32 (57.1%)	90 (46.9%)	0.23
M0 meeting context (n, %)	0			
Consultation		32 (48.5%)	158 (67.5%)	
Hospitalization		34 (51.5%)	90 (46.9%)	**0.0072**
Age at inclusion (y), *mean ± SD*	0	63.6 ± 13.4	61.8 ± 15.7	0.61
Age at gout diagnosis (y), *mean ± SD*	13	59.7 ± 15.5	55 ± 17.6	0.056
Duration of disease before inclusion (y), *mean ± SD*	14	4.2 ± 6.6	6.3 ± 9	**0.02**
Number of gout flares over the last 6 months before inclusion, *mean ± SD*	18	1.9 ± 2.4	2.2 ± 2.8	0.39
M0 uricemia (mg/dL), *mean ± SD*	17	8.09 ± 2.54	8.21 ± 2.33	0.75
Gout complications before M0[a], *mean ± SD*	0	1 ± 0.8	1.2 ± 1	0.21
Extra-rheumatologic comorbidities[b], *mean ± SD*	1	1.8 ± 1.5	1.6 ± 1.4	0.51

[a] Ultrasound or subcutaneous tophi, renal lithiasis on imaging and/or renal colic, chronic kidney disease (CKD 3 and above), gouty arthropathy.

[b] Diabetes mellitus, high blood pressure, history of major cardiovascular event (stroke, myocardial infarction, lower limb arteriopathy), congestive heart failure, dyslipidaemia, liver disease, obesity.
